# 1-Benzoyl-3,5-diphenyl-4,5-dihydro-1*H*-pyrazole

**DOI:** 10.1107/S1600536811003631

**Published:** 2011-02-05

**Authors:** Chang-Zheng Zheng, Liang Wang, Juan Liu

**Affiliations:** a College of Environment and Chemical Engineering, Xi’an Polytechnic University, 710048 Xi’an, Shaanxi, People’s Republic of China

## Abstract

In the title compound, C_22_H_18_N_2_O, the pyrazole ring is almost planar (r.m.s. deviation = 0.0098 Å) and its mean plane makes dihedral angles of 62.2 (2), 87.2 (2) and 8.0 (2)° with the phenyl and benzoyl rings, respectively. The crystal packing is stabilized by π–π stacking inter­actions [centroid–centroid distance = 3.658 (2) Å] and weak inter­molecular C—H⋯O hydrogen bonds.

## Related literature

For the coordination properties of aroylhydrazones, see: Egli *et al.* (2006 ▶); Ge (2006 ▶); Chopra *et al.* (2006 ▶). For related structures, see: Seebacher *et al.* (2003 ▶); Ge (2006 ▶); Jian & Wang (2006 ▶); Fun *et al.* (2010 ▶). For bond-length data, see: Allen *et al.* (1987 ▶).
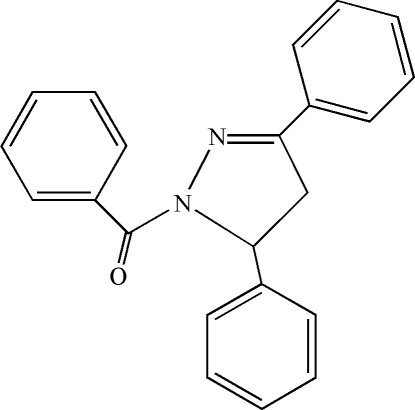


## Experimental

### 

#### Crystal data

C_22_H_18_N_2_O
*M* *_r_* = 326.38
Orthorhombic, 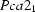

*a* = 20.276 (6) Å
*b* = 5.7859 (17) Å
*c* = 14.786 (4) Å
*V* = 1734.5 (9) Å^3^
*Z* = 4
Mo *K*α radiation
μ = 0.08 mm^−1^
*T* = 298 K
0.18 × 0.16 × 0.12 mm

#### Data collection

Bruker SMART CCD area-detector diffractometer
Absorption correction: multi-scan (*SADABS*; Sheldrick, 2006) ▶ *T*_min_ = 0.986, *T*_max_ = 0.991
8497 measured reflections
1601 independent reflections
1100 reflections with *I* > 2σ(*I*)
*R*_int_ = 0.050

#### Refinement

*R*[*F* ^2^ > 2σ(*F* ^2^)] = 0.035
*wR*(*F* ^2^) = 0.081
*S* = 1.09
1601 reflections
227 parameters
H-atom parameters constrained
Δρ_max_ = 0.13 e Å^−3^
Δρ_min_ = −0.10 e Å^−3^

### 

Data collection: *SMART* (Bruker, 1996 ▶); cell refinement: *SAINT* (Bruker, 1996 ▶); data reduction: *SAINT*; program(s) used to solve structure: *SHELXS97* (Sheldrick, 2008 ▶); program(s) used to refine structure: *SHELXL97* (Sheldrick, 2008 ▶); molecular graphics: *SHELXTL* (Sheldrick, 2008 ▶); software used to prepare material for publication: *SHELXTL*.

## Supplementary Material

Crystal structure: contains datablocks I, global. DOI: 10.1107/S1600536811003631/zq2084sup1.cif

Structure factors: contains datablocks I. DOI: 10.1107/S1600536811003631/zq2084Isup2.hkl

Additional supplementary materials:  crystallographic information; 3D view; checkCIF report

## Figures and Tables

**Table 1:** Hydrogen-bond geometry (Å, °)

*D*—H⋯*A*	*D*—H	H⋯*A*	*D*⋯*A*	*D*—H⋯*A*
C21—H21⋯O1^i^	0.93	2.72	3.399 (5)	131
C22—H22⋯O1^i^	0.93	3.00	3.540 (4)	119
C10—H10⋯O1^ii^	0.93	2.87	3.793 (5)	174

Symmetry codes: (i) 
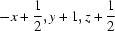
; (ii) 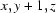.

**Fig. 1.**
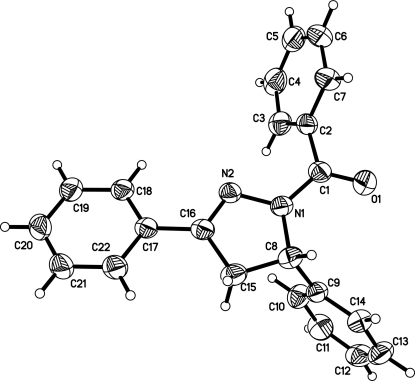
The molecular structure of the title compound, showing displacement ellipsoids drawn at the 30% probability level. H atoms have been omitted for clarity.

**Fig. 2.**
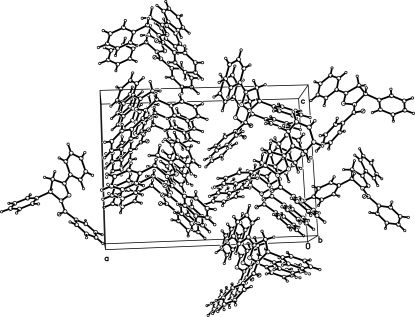
The crystal packing of the title compound.

## supplementary crystallographic information

### Comment

The chemistry of aroylhydrazones continues to attract much attention due to their coordination ability to metal ions (Egli *et al.*, 2006; Ge, 2006) and their biological activity (Egli *et al.*, 2006; Chopra *et al.*, 2006). As an extension of work on the structural characterization of aroylhydrazone derivatives, the title compound,*C*_22_H_18_N_2_O, was successfully synthesized and its crystal structure is reported here.

In the title complex, C_22_H_18_N_2_O, all bond lengths and angles are normal (Allen *et al.*, 1987). The pyrazole ring is planar (rms deviation = 0.0098 Å) and its mean plane makes dihedral angles of 62.2 (1), 87.2 (1) and 8.0 (2)° with the benzene rings C2-C7, C9-C14 and C17-C22, respectively (Fig. 1). The crystal packing is stabilized by π-π stacking interactions between the pyrazole ring and one benzene ring with a centroid-centroid separation of 3.658 (2) Å and by weak intermolecular C—H···O hydrogen bonds (Fig.2; Table 1).

### Experimental

A methanol solution (10 ml) of *N*'-(*E*)-(benzylidene acetophenone phenmethyl acylhydrazone) (0.25 mmol,0.082 g) was mixed with a DMF solution (5 ml). The mixture was stirred at 298 K for 2 h. and then filtered. A colorless precipitate was produced after about 20 days. A DMF amount (5 ml) was used to dissolve the precipitate at 330 K. Colorless block-shaped crystals of the title complex were obtained after one month (yield 30%).

### Refinement

H atoms were placed in calculated positions and refined as riding with the following constraints: C-H = 0.93 Å and *U*_iso_(H) = 1.2*U*_eq_(C) for aromatic H atoms, C-H = 0.97 Å and *U*_iso_(H) = 1.2*U*_eq_(C) for methylene H atoms, and C-H = 0.98 Å and *U*_iso_(H) = 1.2*U*_eq_(C) for methine H atoms. As the structure has no anomalous scatterer, the Friedel-pair reflections were merged.

### Crystal data

**Table:**

C_22_H_18_N_2_O	*F*(000) = 688
*M**_r_* = 326.38	*D*_x_ = 1.250 Mg m^−^^3^
Orthorhombic, *P**c**a*2_1_	Mo *K*α radiation, λ = 0.71073 Å
Hall symbol: P 2c -2ac	Cell parameters from 1072 reflections
*a* = 20.276 (6) Å	θ = 2.4–17.6°
*b* = 5.7859 (17) Å	µ = 0.08 mm^−^^1^
*c* = 14.786 (4) Å	*T* = 298 K
*V* = 1734.5 (9) Å^3^	Block, colorless
*Z* = 4	0.18 × 0.16 × 0.12 mm

### Data collection

**Table:**

Bruker SMART CCD area-detector diffractometer	1601 independent reflections
Radiation source: fine-focus sealed tube	1100 reflections with *I* > 2σ(*I*)
graphite	*R*_int_ = 0.050
φ and ω scans	θ_max_ = 25.1°, θ_min_ = 2.0°
Absorption correction: multi-scan (*SADABS*; Sheldrick, 2006)	*h* = −24→23
*T*_min_ = 0.986, *T*_max_ = 0.991	*k* = −6→6
8497 measured reflections	*l* = −17→13

### Refinement

**Table:**

Refinement on *F*^2^	Secondary atom site location: difference Fourier map
Least-squares matrix: full	Hydrogen site location: inferred from neighbouring sites
*R*[*F*^2^ > 2σ(*F*^2^)] = 0.035	H-atom parameters constrained
*wR*(*F*^2^) = 0.081	*w* = 1/[σ^2^(*F*_o_^2^) + (0.0335*P*)^2^] where *P* = (*F*_o_^2^ + 2*F*_c_^2^)/3
*S* = 1.09	(Δ/σ)_max_ < 0.001
1601 reflections	Δρ_max_ = 0.13 e Å^−^^3^
227 parameters	Δρ_min_ = −0.10 e Å^−^^3^
0 restraints	Extinction correction: *SHELXL97* (Sheldrick, 2008)
Primary atom site location: structure-invariant direct methods	Extinction coefficient: 0.0113 (15)

### Special details

**Table:**

Geometry. All e.s.d.'s (except the e.s.d. in the dihedral angle between two l.s. planes) are estimated using the full covariance matrix. The cell e.s.d.'s are taken into account individually in the estimation of e.s.d.'s in distances, angles and torsion angles; correlations between e.s.d.'s in cell parameters are only used when they are defined by crystal symmetry. An approximate (isotropic) treatment of cell e.s.d.'s is used for estimating e.s.d.'s involving l.s. planes.
Refinement. Refinement of *F*^2^ against ALL reflections. The weighted *R*-factor *wR* and goodness of fit *S* are based on *F*^2^, conventional *R*-factors *R* are based on *F*, with *F* set to zero for negative *F*^2^. The threshold expression of *F*^2^ > σ(*F*^2^) is used only for calculating *R*-factors(gt) *etc*. and is not relevant to the choice of reflections for refinement. *R*-factors based on *F*^2^ are statistically about twice as large as those based on *F*, and *R*- factors based on ALL data will be even larger.

### Fractional atomic coordinates and isotropic or equivalent isotropic displacement parameters (Å^2^)

**Table:**

	*x*	*y*	*z*	*U*_iso_*/*U*_eq_	
O1	0.27196 (11)	0.5466 (4)	0.77340 (17)	0.0769 (7)	
N1	0.28823 (12)	0.8795 (5)	0.84788 (17)	0.0617 (7)	
N2	0.33000 (12)	1.0610 (4)	0.87296 (18)	0.0587 (7)	
C1	0.30374 (16)	0.7269 (6)	0.7810 (2)	0.0607 (8)	
C2	0.35797 (16)	0.7877 (6)	0.7181 (2)	0.0602 (8)	
C3	0.36177 (18)	0.9982 (7)	0.6747 (3)	0.0742 (10)	
H3	0.3325	1.1160	0.6895	0.089*	
C4	0.4093 (2)	1.0336 (9)	0.6092 (3)	0.0904 (12)	
H4	0.4105	1.1731	0.5780	0.109*	
C5	0.4545 (2)	0.8659 (11)	0.5897 (3)	0.1057 (16)	
H5	0.4870	0.8929	0.5465	0.127*	
C6	0.4521 (2)	0.6593 (10)	0.6336 (3)	0.1046 (16)	
H6	0.4833	0.5458	0.6209	0.125*	
C7	0.40374 (18)	0.6183 (7)	0.6965 (3)	0.0840 (11)	
H7	0.4016	0.4752	0.7250	0.101*	
C8	0.23482 (15)	0.8362 (6)	0.9137 (2)	0.0630 (9)	
H8	0.2392	0.6799	0.9386	0.076*	
C9	0.16756 (15)	0.8651 (6)	0.8719 (2)	0.0566 (8)	
C10	0.15150 (17)	1.0621 (6)	0.8242 (3)	0.0719 (10)	
H10	0.1833	1.1755	0.8150	0.086*	
C11	0.0887 (2)	1.0930 (7)	0.7897 (3)	0.0838 (11)	
H11	0.0788	1.2254	0.7568	0.101*	
C12	0.04134 (19)	0.9293 (9)	0.8040 (3)	0.0874 (12)	
H12	−0.0014	0.9531	0.7830	0.105*	
C13	0.05668 (19)	0.7314 (8)	0.8490 (3)	0.0860 (12)	
H13	0.0248	0.6176	0.8568	0.103*	
C14	0.11969 (17)	0.6987 (6)	0.8833 (3)	0.0726 (10)	
H14	0.1297	0.5632	0.9142	0.087*	
C15	0.25078 (16)	1.0154 (6)	0.9873 (2)	0.0690 (9)	
H15A	0.2141	1.1206	0.9964	0.083*	
H15B	0.2612	0.9407	1.0443	0.083*	
C16	0.30982 (15)	1.1402 (5)	0.9498 (2)	0.0572 (8)	
C17	0.34201 (15)	1.3367 (6)	0.9937 (2)	0.0578 (8)	
C18	0.39084 (15)	1.4622 (6)	0.9502 (3)	0.0655 (9)	
H18	0.4046	1.4170	0.8929	0.079*	
C19	0.41933 (17)	1.6525 (6)	0.9903 (3)	0.0745 (10)	
H19	0.4518	1.7353	0.9598	0.089*	
C20	0.39988 (19)	1.7206 (7)	1.0755 (3)	0.0785 (11)	
H20	0.4191	1.8490	1.1027	0.094*	
C21	0.3520 (2)	1.5976 (7)	1.1199 (3)	0.0802 (11)	
H21	0.3391	1.6421	1.1777	0.096*	
C22	0.32291 (17)	1.4088 (6)	1.0798 (2)	0.0718 (10)	
H22	0.2901	1.3283	1.1104	0.086*	

### Atomic displacement parameters (Å^2^)

**Table:**

	*U*^11^	*U*^22^	*U*^33^	*U*^12^	*U*^13^	*U*^23^
O1	0.0671 (16)	0.0745 (15)	0.0890 (18)	−0.0040 (13)	−0.0021 (13)	−0.0112 (14)
N1	0.0474 (15)	0.0756 (18)	0.0622 (18)	0.0008 (14)	0.0018 (13)	−0.0107 (15)
N2	0.0485 (14)	0.0682 (18)	0.0594 (17)	0.0074 (14)	−0.0021 (13)	−0.0077 (15)
C1	0.049 (2)	0.070 (2)	0.062 (2)	0.0125 (18)	−0.0095 (17)	−0.008 (2)
C2	0.056 (2)	0.074 (2)	0.051 (2)	0.0028 (18)	−0.0054 (16)	−0.0150 (19)
C3	0.066 (2)	0.094 (3)	0.063 (2)	−0.002 (2)	−0.0115 (19)	−0.007 (2)
C4	0.094 (3)	0.116 (3)	0.062 (2)	−0.027 (3)	−0.002 (2)	−0.010 (2)
C5	0.090 (3)	0.149 (4)	0.077 (3)	−0.039 (4)	0.024 (3)	−0.051 (3)
C6	0.084 (3)	0.121 (4)	0.109 (4)	−0.002 (3)	0.027 (3)	−0.055 (3)
C7	0.073 (3)	0.095 (3)	0.083 (3)	0.004 (2)	0.012 (2)	−0.024 (2)
C8	0.052 (2)	0.073 (2)	0.064 (2)	0.0006 (17)	0.0000 (16)	0.0051 (18)
C9	0.0491 (17)	0.064 (2)	0.057 (2)	−0.0009 (16)	0.0016 (15)	−0.0036 (18)
C10	0.062 (2)	0.075 (2)	0.079 (2)	−0.0017 (19)	−0.0084 (18)	0.001 (2)
C11	0.078 (3)	0.091 (3)	0.083 (3)	0.015 (2)	−0.020 (2)	−0.002 (2)
C12	0.053 (2)	0.123 (3)	0.086 (3)	0.012 (3)	−0.007 (2)	−0.015 (3)
C13	0.057 (2)	0.116 (4)	0.085 (3)	−0.022 (2)	0.006 (2)	−0.013 (3)
C14	0.063 (2)	0.078 (3)	0.077 (2)	−0.007 (2)	0.0091 (19)	−0.002 (2)
C15	0.0513 (18)	0.100 (2)	0.056 (2)	−0.0009 (19)	0.0007 (16)	−0.003 (2)
C16	0.0480 (18)	0.075 (2)	0.0483 (19)	0.0102 (16)	−0.0053 (15)	−0.0023 (18)
C17	0.0468 (18)	0.078 (2)	0.0482 (19)	0.0109 (17)	−0.0077 (16)	−0.0046 (17)
C18	0.052 (2)	0.089 (2)	0.0554 (19)	0.0029 (18)	−0.0022 (18)	−0.008 (2)
C19	0.063 (2)	0.091 (3)	0.070 (3)	−0.0057 (19)	−0.0032 (19)	−0.007 (2)
C20	0.071 (3)	0.090 (3)	0.075 (3)	0.008 (2)	−0.018 (2)	−0.020 (2)
C21	0.077 (3)	0.103 (3)	0.060 (2)	0.004 (2)	−0.005 (2)	−0.019 (2)
C22	0.068 (2)	0.098 (3)	0.049 (2)	0.0001 (19)	−0.0013 (17)	−0.006 (2)

### Geometric parameters (Å, °)

**Table:**

O1—C1	1.231 (4)	C10—H10	0.9300
N1—C1	1.362 (4)	C11—C12	1.365 (5)
N1—N2	1.399 (3)	C11—H11	0.9300
N1—C8	1.478 (4)	C12—C13	1.360 (5)
N2—C16	1.292 (4)	C12—H12	0.9300
C1—C2	1.482 (4)	C13—C14	1.387 (5)
C2—C3	1.379 (5)	C13—H13	0.9300
C2—C7	1.387 (4)	C14—H14	0.9300
C3—C4	1.381 (5)	C15—C16	1.504 (5)
C3—H3	0.9300	C15—H15A	0.9700
C4—C5	1.366 (6)	C15—H15B	0.9700
C4—H4	0.9300	C16—C17	1.463 (4)
C5—C6	1.361 (6)	C17—C18	1.386 (4)
C5—H5	0.9300	C17—C22	1.394 (4)
C6—C7	1.373 (6)	C18—C19	1.378 (5)
C6—H6	0.9300	C18—H18	0.9300
C7—H7	0.9300	C19—C20	1.378 (5)
C8—C9	1.507 (4)	C19—H19	0.9300
C8—C15	1.537 (4)	C20—C21	1.371 (5)
C8—H8	0.9800	C20—H20	0.9300
C9—C14	1.377 (4)	C21—C22	1.377 (5)
C9—C10	1.380 (4)	C21—H21	0.9300
C10—C11	1.383 (5)	C22—H22	0.9300
			
C1—N1—N2	122.6 (3)	C12—C11—H11	120.0
C1—N1—C8	122.5 (3)	C10—C11—H11	120.0
N2—N1—C8	113.3 (3)	C13—C12—C11	119.9 (4)
C16—N2—N1	107.9 (3)	C13—C12—H12	120.0
O1—C1—N1	119.6 (3)	C11—C12—H12	120.0
O1—C1—C2	122.1 (3)	C12—C13—C14	120.3 (4)
N1—C1—C2	118.2 (3)	C12—C13—H13	119.9
C3—C2—C7	118.7 (3)	C14—C13—H13	119.9
C3—C2—C1	122.9 (3)	C9—C14—C13	120.6 (4)
C7—C2—C1	118.2 (3)	C9—C14—H14	119.7
C2—C3—C4	119.7 (4)	C13—C14—H14	119.7
C2—C3—H3	120.1	C16—C15—C8	103.3 (3)
C4—C3—H3	120.1	C16—C15—H15A	111.1
C5—C4—C3	120.7 (4)	C8—C15—H15A	111.1
C5—C4—H4	119.6	C16—C15—H15B	111.1
C3—C4—H4	119.6	C8—C15—H15B	111.1
C6—C5—C4	120.0 (4)	H15A—C15—H15B	109.1
C6—C5—H5	120.0	N2—C16—C17	121.6 (3)
C4—C5—H5	120.0	N2—C16—C15	114.0 (3)
C5—C6—C7	120.0 (4)	C17—C16—C15	124.4 (3)
C5—C6—H6	120.0	C18—C17—C22	117.7 (3)
C7—C6—H6	120.0	C18—C17—C16	121.4 (3)
C6—C7—C2	120.8 (4)	C22—C17—C16	120.9 (3)
C6—C7—H7	119.6	C19—C18—C17	121.3 (3)
C2—C7—H7	119.6	C19—C18—H18	119.4
N1—C8—C9	112.0 (2)	C17—C18—H18	119.4
N1—C8—C15	101.4 (3)	C18—C19—C20	120.1 (4)
C9—C8—C15	114.0 (3)	C18—C19—H19	119.9
N1—C8—H8	109.7	C20—C19—H19	119.9
C9—C8—H8	109.7	C21—C20—C19	119.5 (4)
C15—C8—H8	109.7	C21—C20—H20	120.2
C14—C9—C10	118.2 (3)	C19—C20—H20	120.2
C14—C9—C8	120.7 (3)	C20—C21—C22	120.6 (4)
C10—C9—C8	121.0 (3)	C20—C21—H21	119.7
C9—C10—C11	120.8 (4)	C22—C21—H21	119.7
C9—C10—H10	119.6	C21—C22—C17	120.8 (4)
C11—C10—H10	119.6	C21—C22—H22	119.6
C12—C11—C10	120.1 (4)	C17—C22—H22	119.6
			
C1—N1—N2—C16	−165.4 (3)	C14—C9—C10—C11	0.9 (5)
C8—N1—N2—C16	0.8 (3)	C8—C9—C10—C11	−176.8 (3)
N2—N1—C1—O1	166.0 (3)	C9—C10—C11—C12	1.1 (6)
C8—N1—C1—O1	1.0 (4)	C10—C11—C12—C13	−2.7 (6)
N2—N1—C1—C2	−15.4 (4)	C11—C12—C13—C14	2.3 (6)
C8—N1—C1—C2	179.6 (3)	C10—C9—C14—C13	−1.3 (5)
O1—C1—C2—C3	128.9 (3)	C8—C9—C14—C13	176.4 (3)
N1—C1—C2—C3	−49.7 (4)	C12—C13—C14—C9	−0.3 (6)
O1—C1—C2—C7	−45.7 (4)	N1—C8—C15—C16	2.1 (3)
N1—C1—C2—C7	135.7 (3)	C9—C8—C15—C16	−118.4 (3)
C7—C2—C3—C4	2.0 (5)	N1—N2—C16—C17	−178.2 (2)
C1—C2—C3—C4	−172.5 (3)	N1—N2—C16—C15	0.8 (3)
C2—C3—C4—C5	−3.0 (5)	C8—C15—C16—N2	−1.9 (4)
C3—C4—C5—C6	1.5 (6)	C8—C15—C16—C17	177.0 (3)
C4—C5—C6—C7	0.8 (7)	N2—C16—C17—C18	7.1 (4)
C5—C6—C7—C2	−1.8 (6)	C15—C16—C17—C18	−171.7 (3)
C3—C2—C7—C6	0.3 (5)	N2—C16—C17—C22	−175.0 (3)
C1—C2—C7—C6	175.2 (3)	C15—C16—C17—C22	6.2 (4)
C1—N1—C8—C9	−73.8 (4)	C22—C17—C18—C19	−0.4 (5)
N2—N1—C8—C9	120.0 (3)	C16—C17—C18—C19	177.5 (3)
C1—N1—C8—C15	164.3 (3)	C17—C18—C19—C20	0.6 (5)
N2—N1—C8—C15	−1.9 (3)	C18—C19—C20—C21	0.0 (5)
N1—C8—C9—C14	131.1 (3)	C19—C20—C21—C22	−0.7 (5)
C15—C8—C9—C14	−114.5 (3)	C20—C21—C22—C17	0.9 (5)
N1—C8—C9—C10	−51.3 (4)	C18—C17—C22—C21	−0.3 (5)
C15—C8—C9—C10	63.1 (4)	C16—C17—C22—C21	−178.2 (3)

### Hydrogen-bond geometry (Å, °)

**Table:**

*D*—H···*A*	*D*—H	H···*A*	*D*···*A*	*D*—H···*A*
C21—H21···O1^i^	0.93	2.72	3.399 (5)	131
C22—H22···O1^i^	0.93	3.00	3.540 (4)	119
C10—H10···O1^ii^	0.93	2.87	3.793 (5)	174

Symmetry codes: (i) −*x*+1/2, *y*+1, *z*+1/2; (ii) *x*, *y*+1, *z*.
